# The nomogram to predict the occurrence of sepsis-associated encephalopathy in elderly patients in the intensive care units: A retrospective cohort study

**DOI:** 10.3389/fneur.2023.1084868

**Published:** 2023-02-02

**Authors:** Qing Zhao, Jianguo Xiao, Xiaoli Liu, Hui Liu

**Affiliations:** ^1^Department of Diagnosis and Treatment of Cadres, First Medical Center, Chinese PLA General Hospital, Beijing, China; ^2^Department of Critical Care Medicine, The First Medical Center, Chinese PLA General Hospital, Beijing, China; ^3^Key Laboratory for Biomechanics and Mechanobiology of Ministry of Education, Beijing Advanced Innovation Center for Biomedical Engineering, School of Biological Science and Medical Engineering, Beihang University, Beijing, China

**Keywords:** SAE, nomogram, elderly patients, mortality, SOFA

## Abstract

**Background:**

Sepsis-associated encephalopathy (SAE) is a critical and common problem in elderly patients with sepsis, which is still short of efficient predictive tools. Therefore, this study aims to screen the risk factors and establish a useful predictive nomogram for SAE in elderly patients with sepsis in the intensive care unit (ICU).

**Patients and methods:**

Elderly patients (age ≥ 65 years) with sepsis were selected from the Medical Information Mart for Intensive Care (MIMIC)-IV database. Data from demographics and laboratory examinations were collected on the first day of admission to the ICU. SAE was defined by two criteria in the presence of sepsis: ① a Glasgow Coma Scale (GCS) score of < 15 or ② delirium. Differences in demographics and laboratory tests were calculated between SAE and non-SAE groups. Participants were randomly divided into a training set and a validation set without replacement at a ratio of 6:4. A predictive nomogram was constructed in the training set by logistic regression analysis and then validated. The predictive capability of the nomogram was demonstrated by receiver operating characteristic (ROC) analysis and calibration curve analysis.

**Results:**

A total of 22,361 patients were selected, of which 2,809 patients (12.7%) died in the hospital and 8,290 patients (37.1%) had SAE. In-hospital mortality in the SAE group was higher than that in the non-SAE group (18.8 vs. 8.9%, *p* < 0.001). Based on the results of logistic regression analysis, a nomogram integrating age, Na^+^, Sequential Organ Failure Assessment (SOFA) score, heart rate, and body temperature were constructed. The area under the curve (AUC) of the nomogram was 80.2% in the training set and 80.9% in the validation set. Calibration curve analysis showed a good predictive capacity of the nomogram.

**Conclusion:**

SAE is an independent risk of in-hospital mortality in elderly patients in the intensive care unit. The nomogram has an excellent predictive capability of SAE and helps in clinical practice.

## 1. Introduction

Sepsis-associated encephalopathy (SAE) is a critical complication of patients with sepsis, especially in the elderly population. The clinical manifestation of SAE comprises impairment of awareness, delirium, and coma (1). Patients with SAE suffer from deteriorative cognitive function, bad memory, attention loss, and a decrease in verbal fluency. Sepsis-related delirium is mostly hypoactive, and only a few cases are hyperactive or mixed. Hypoactive delirium is characterized by slowed motor function, confusion, sedation, impaired response, withdrawn attitude, and drowsiness. Hyperactive delirium is commonly characterized by agitation, emotional lability, restlessness, and other positive psychotic manifestations. Patients with sepsis are weak with severe complications, such as shock, infection, and hypoxia. Sedatives are often used in critical patients with sepsis, which also lead to hypoactive delirium. Hypoactive delirium is more often observed in patients with sepsis than in hyperactive delirium. The exacerbation of SAE is associated with focal deficits, seizures, tremors, or nonepileptic myoclonus (2–4). Psychological disorders are common in patients with SAE, such as anxiety or post-traumatic stress disorder (PTSD) (5, 6). SAE is associated with increased mortality and poor long-term outcomes. Its incidence varies from no more than 10% to over 70% according to the severity of sepsis in critical patients in the ICU (7). Early recognition of risk factors related to SAE is important for timely clinical treatment. Elderly patients are more vulnerable to the dysfunction of the central nervous system and develop SAE. However, there are few studies on the epidemic of SAE and relevant risk factors in the elderly population. In this study, we examined the morbidity of SAE and developed an SAE prediction model in critical elderly patients.

## 2. Patients and methods

### 2.1. Study design and participants' enrollment

**Figure 1:**
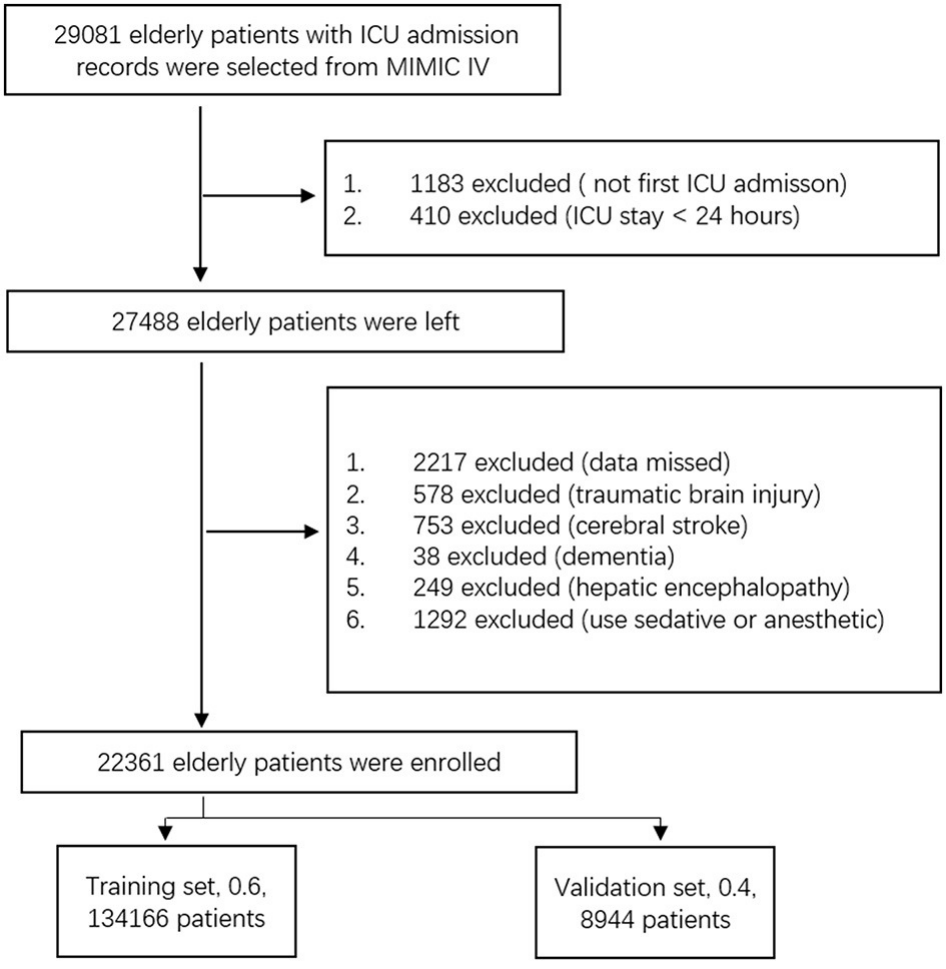
Flow of enrollment.

This was a retrospective cohort study. In this retrospective study, we selected elderly (≥65 years) patients between 2008 and 2019 from the MIMIC-IV database (8, 9). Demographics and laboratory examination data of elderly patients (≥ 65 years) were collected on the first day of ICU admission. The incidence of SAE was demonstrated. Risk factors of SAE were screened, and the nomogram model was established. Enrolled patients were divided into a training set and a validation set. The efficiency of the nomogram was validated. The inclusion criteria were as follows: (1) age ≥ 65 years, (2) first admission to the ICU, and (3) a length of ICU stay of ≥ 24 h. The exclusion criteria were as follows: (1) data missing, without records of delirium and Glasgow score; (2) traumatic brain injury; (3) cerebral stroke; (4) dementia; (5) hepatic encephalopathy, and (6) use of sedatives or anesthetics. The enrollment workflow is shown in Figure 1.

### 2.2. Terms and definitions

- Sepsis: Organ dysfunction, hypoperfusion, or hypotension caused by systemic infection based upon the sepsis 3.0 definition. Patients with infection and a SOFA score of ≥2 were regarded as cases with sepsis 3.0 (11).
- Delirium: Patients were identified using the Richmond Agitation Sedation Scale (RASS) and the confusion assessment method for the intensive care unit (CAM-ICU) (12). RASS is graded into unarousable, deep sedation, moderate sedation, light sedation, drowsy, alert and calm, restless, agitated, very agitated, and combative, which are numbered between −5 and +4. According to the CAM-ICU procedure, delirium is confirmed if (i) the RASS score is ≥ −3; (ii) there is an acute change in the mental status baseline; (iii) there are >2 errors in the attention test; and (iv) either the RASS score is not 0, or the combined number of errors in the cognitive test is >1.
- SAE: Patients with sepsis who met the inclusion and exclusion criteria were screened. Patients with sepsis who had a Glasgow Coma Scale (GCS) score of < 15 or had manifestations of delirium (diagnosed by the method mentioned earlier) were identified as having SAE (10, 13, 14). GCS is a common tool for assessing the severity of brain injury. The GCS score ranges from 3 to 15. A GCS score ≤ 8 indicates severe brain injury, th GCS scores of 9–12 represent moderate injury, and a GCS score ≥13 represents mild injury.
- Shock: Shock was assumed when any vasopressor therapy was administered, which included norepinephrine, epinephrine, phenylephrine, vasopressin, dopamine, dobutamine, and milrinone.

### 2.3. Baseline data and outcome data

Baseline data were collected on the first day of ICU admission, including sex, age, hemoglobin, white blood cell (WBC), platelet count, creatinine, blood glucose, Na^+^, oxygen pressure of artery (PaO_2_), heart rate (HR), mean artery pressure (MAP), respiratory rate (RR), body temperature, Sequential Organ Failure Assessment (SOFA) score, diagnosis of sepsis, diagnosis of delirium, and diagnosis of shock. Outcome data included in-hospital mortality, length of hospital stay, and length of ICU stay.

### 2.4. Source of data and ethics approval

Medical Information Mart for Intensive Care IV (MIMIC-IV) is an updated version of MIMIC-III with approval from the institutional review board. MIMIC-IV remarkably evolves in structure, includes new data elements, and improves the usability of previous data elements. Up to now, the clinical data in the MIMIC-IV between 2008 and 2019 contain comprehensive and high-quality data of patients admitted to intensive care units (ICUs) at the Beth Israel Deaconess Medical Center. One author (XLL) obtained access to the database and was responsible for data extraction. This project was approved by the Research Ethics Committee of Chinese PLA General Hospital (S2017–054–01).

### 2.5. Statistics

R version 4.1.1 was used. The continuous variables were expressed as mean ± standard deviation (SD). Enumeration data were expressed as numbers and percentages. Categorical variables were analyzed using the odds ratio (OR) and the Chi-square (χ^2^) test. Two groups of continuous variables were assessed using the Mann–Whitney *U*-test. The Kaplan–Meier survival analysis and the log-rank test were used to demonstrate the mortality in elderly patients with or without SAE. The determinant of SAE was evaluated using logistic regression analyses through independent variables. The risk factors in the nomogram were chosen based on the results of logistic regression analysis, previous comparable reports, and clinical knowledge of medicine. Enrolled participants were divided into a training set and a validation set in a ratio of 6:4. All verifications of the nomogram were performed in both the training set and the validation set. Receiver operator characteristic (ROC) curve analysis was used to demonstrate the predictive capacity of the nomogram. Calibration curve analysis and decision curve analysis were also implemented. All statistical tests were two-sided and a *p*-value of < 0.05 was considered statistically significant.

## 3. Results

### 3.1. Demographic and clinical data of the enrolled patients

A total of 22,361 elderly patients (≥ 65 years) in the intensive unit were enrolled, of which 2,809 patients died in the hospital (the mortality was 12.7%). The proportion of patients with SAE was approximately 37.1% (8,290 persons). Between the SAE and the non-SAE groups, there was a significant difference in age, WBC, platelet, glucose, Na^+^, PaO_2_, heart rate, MBP, respiratory rate, delirium, hemoglobin, SOFA, sepsis, shock, and creatinine. The SOFA of the SAE group was 6.64 ± 3.32, and the SOFA of the non-SAE group was 4.26 ± 3.07. The difference was significant (*p* = 0.000). Compared with patients without SAE, patients with SAE had a longer length of ICU stay (4.94 ± 5.50 vs. 3.30 ± 4.30 days, *p* < 0.001) and hospital stay (12.05 ± 12.37 vs. 9.06 ± 8.97 days, *p* < 0.001). As data shown in Table 1, there were 8,290 patients with SAE and 10,694 patients with sepsis. The incidence of SAE in patients with sepsis was 77.5%.

**Table 1:** Baseline characteristics and outcome data in SAE and non-SAE groups.

**Characteristics**	**All**	**Non-SAE**	**SAE**	***p*** **-value**
Number	22,361	14,071	8,290	
Age (year)	77.25 ± 8.05	76.91 ± 7.99	77.93 ± 8.12	0.000^**^
Female/male	10,341/12,020	6,478/7,593	3,863/4,427	0.425
Hemoglobin (g/dL)	10.05 ± 2.11	10.19 ± 2.14	9.79 ± 2.02	0.000^**^
WBC (10^9^/L)	14.28 ± 11.83	13.47 ± 10.70	15.91 ± 13.69	0.000^**^
Platelet (10^9^/L)	186.92 ± 94.91	187.94 ± 91.63	184.87 ± 101.14	0.023^*^
Creatinine (mg/mL)	1.49 ± 1.35	1.38 ± 1.28	1.70 ± 1.46	0.000^**^
Glucose (mg/dL)	165.10 ± 91.30	159.21 ± 81.24	176.91 ± 107.77	0.000^**^
Na^+^ (mmol/L)	139.81 ± 4.95	139.51 ± 4.52	140.42 ± 5.66	0.000^**^
PaO_2_ (mmHg)	115.13 ± 50.74	117.55 ± 51.37	110.26 ± 49.10	0.000^**^
Heart rate (beat/minute)	82.40 ± 14.93	81.09 ± 14.46	85.01 ± 15.50	0.000^**^
Mean artery pressure (mmHg)	76.89 ± 10.20	77.79 ± 10.46	75.11 ± 9.40	0.000^**^
Respiratory rate (time/minute)	19.17 ± 3.57	18.93 ± 3.42	19.66 ± 3.81	0.000^**^
Body temperature (°C)	36.77 ± 0.48	36.75 ± 0.45	36.83 ± 0.53	0.000^**^
SOFA	5.04 ± 3.35	4.26 ± 3.07	6.64 ± 3.32	0.000^**^
Sepsis (count/percent)	10,694/47.8%	2,404/17.1%	8,290/100%	0.000^**^
Delirium (number/percent)	5,453/24.4%	2,248/16.0%	3,205/38.7%	0.000^**^
Shock (count/percent)	4,370/19.5%	1,767/12.6%	2,603/31.4%	0.000^**^
**Outcome data**				
ICU days (day)	3.85 ± 4.79	3.30 ± 4.30	4.94 ± 5.50	0.000^**^
Hospital days (day)	10.06 ± 10.32	9.06 ± 8.97	12.05 ± 12.37	0.000^**^
In-hospital mortality (count/percent)	2,809/12.7%	1,248/8.9%	1,561/18.8%	0.000^**^

* *p* < 0.05,

** *p* < 0.01.

The values were shown as mean ± SD.

### 3.2. SAE was an independent risk factor of mortality in elderly patients in the ICU

**Figure 2:**
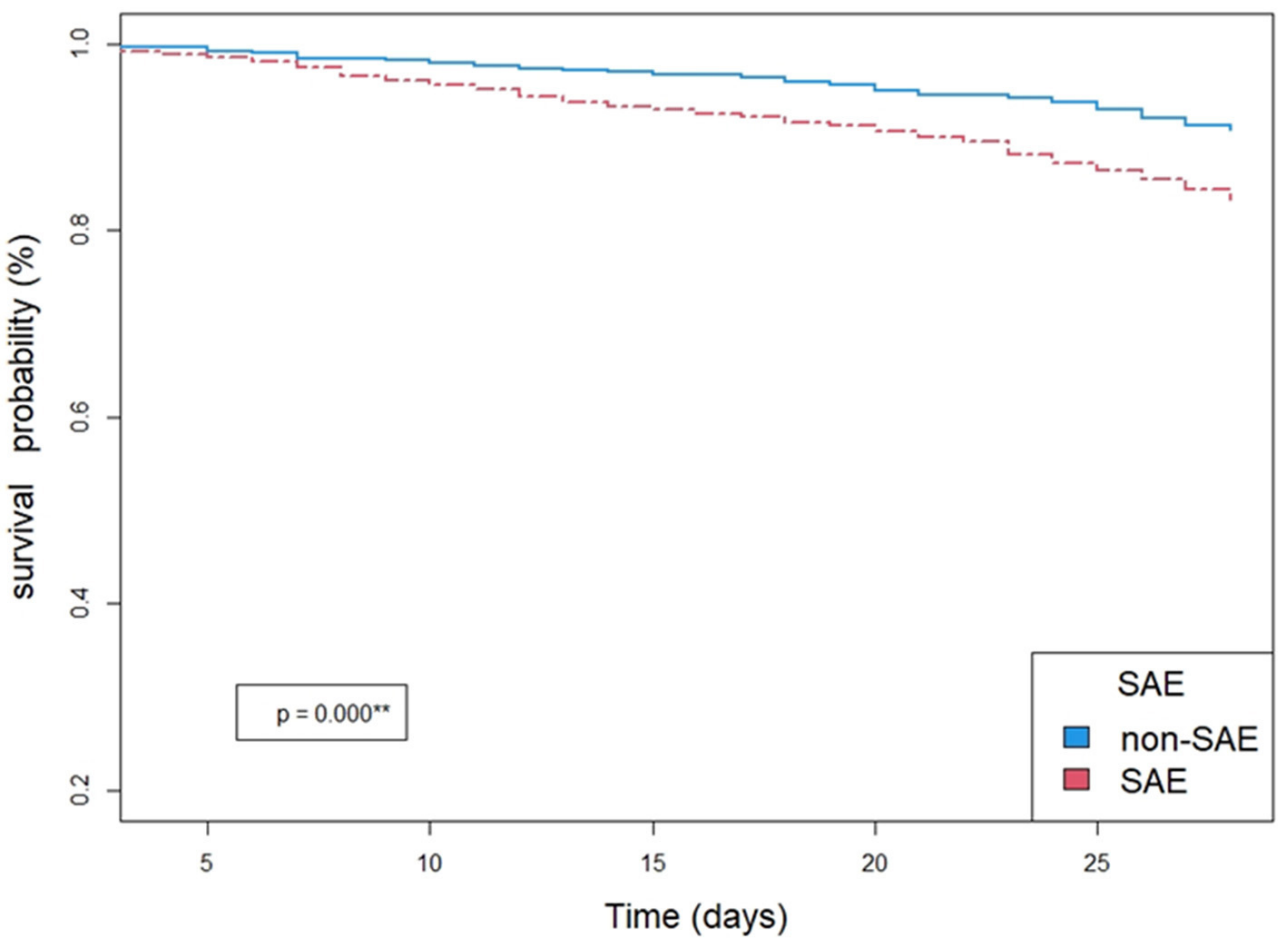
Kaplan-meier survival analysis between groups of SAE and non-SAE.

A total of 18.8% of patients died in the SAE group, which was significantly higher than that (8.9%) in the non-SAE group (*p* < 0.001^**^) (Table 1). To confirm this, the Kaplan–Meier survival analysis was applied to demonstrate the difference in mortality between SAE and non-SAE groups. The results showed that there were more patients who survived in the non-SAE group. The survival probability in non-SAE was significantly higher than that in the SAE group (*p* < 0.001^**^) (Figure 2).

### 3.3. Logistic regression analysis to explore risk factors of SAE

Based on the results of univariable analysis (Table 1), logistic regression analysis was used to identify risk factors of SAE. Sepsis and delirium were not included in the logistic analysis because they were involved in the definition of SAE. Shock and creatinine were excluded because of their collinearity effect with SOFA. SOFA was selected in the logistic regression analysis. Results showed that the significant risk factors were age, SOFA, platelet, Na^+^, heart rate, respiratory rate, and temperature (*p* < 0.05) (Table 2).

**Table 2:** Logistic regression analysis of risk factors for SAE.

**Variable**	**β**	**OR**	**95% CI**	**Z-value**	***p***
Gender	−0.058	0.943	−0.121–0.004	−1.838	0.066
Age	0.018	1.018	0.014–0.022	9.185	0.000^**^
SOFA	0.220	1.246	0.209–0.231	38.562	0.000^**^
Platelet	0.002	1.001	0.001–0.002	9.610	0.000^**^
WBC	0.002	0.998	−0.007–0.002	−1.010	0.313
Glucose	0.001	1.001	0.000–0.001	1.705	0.089
Hemoglobin	−0.015	0.985	−0.031–0.000	−1.932	0.053
Na^+^	0.027	1.028	0.021–0.034	8.549	0.000^**^
PaO_2_	−0.001	1.000	−0.001–0.000	−0.925	0.355
Heart rate	0.011	1.011	0.008–0.013	9.215	0.000^**^
MBP	−0.002	1.002	−0.003–0.007	0.671	0.502
Respiratory rate	−0.014	0.986	−0.027–−0.002	−2.194	0.028^*^
Temperature	0.380	1.462	0.316–0.444	11.713	0.000^**^

* *p* < 0.05,

** *p* < 0.01.

OR, odds ratio; CI, confidence interval.

### 3.4. Development of the prediction nomogram

**Figure 3:**
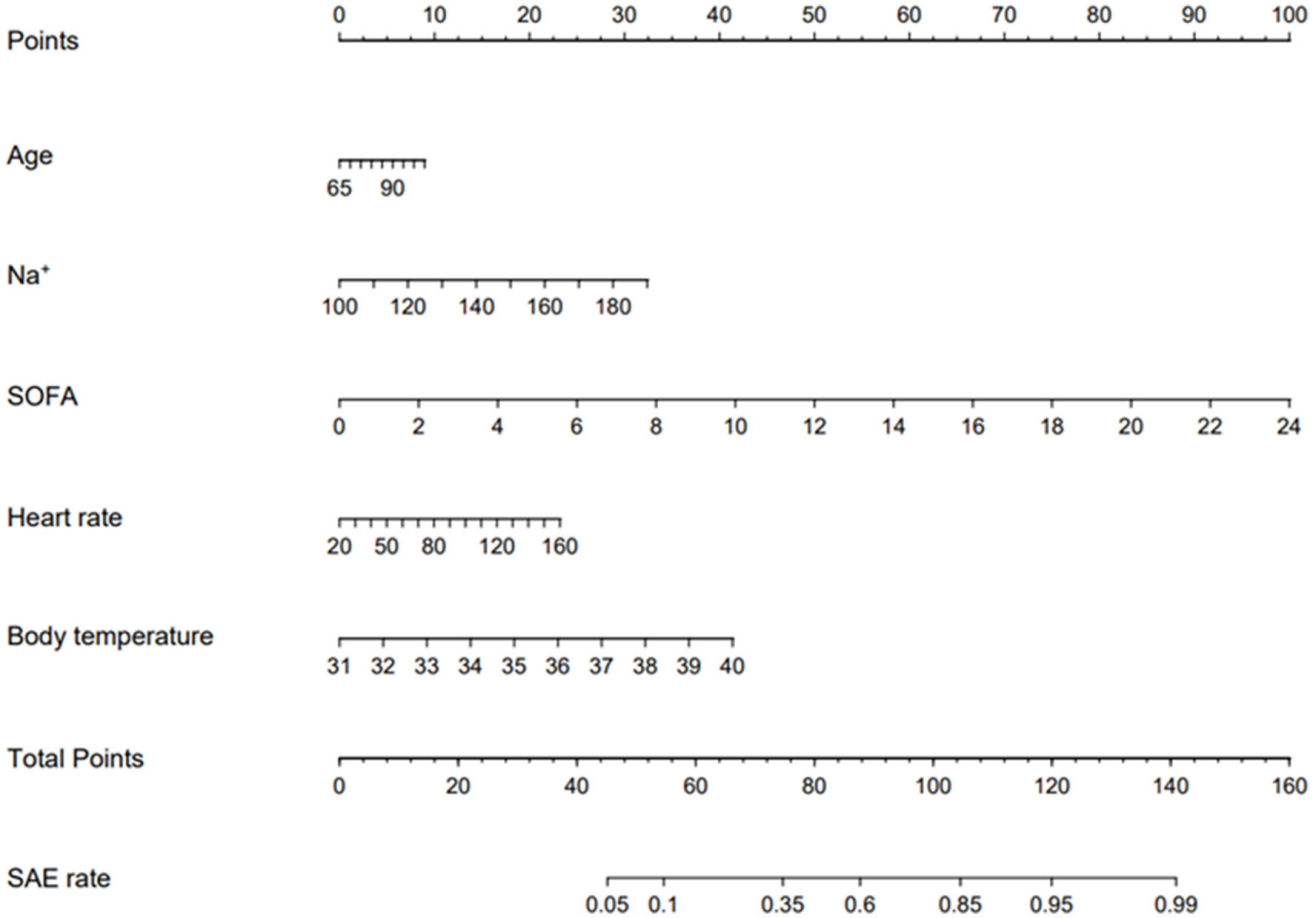
Validated nomogram for predicting 30-day mortality of SAE. When using it, drawing a vertical line from each variables upward to the points and then recording the corresponding points (i.e., “SOFA= 10” = 40 points). The point of each variable was then summed up to obtain a total points that corresponds to a predicted probability of SAE at the bottom of the nomogram.

**Figure 4:**
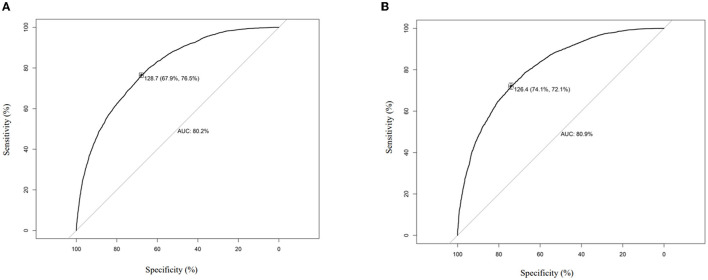
ROC analysis of the nomogram. **(A)** In training set, **(B)** in validation set.

The enrolled participants were divided into a training set and a validation set in a ratio of 6:4. Baseline characteristics between these two sets were demonstrated to be statistically similar (*p* < 0.05) (Supplementary material 1). The risk factors related to SAE are shown in Table 2. Platelet and respiratory rate were removed due to their weak impact on SAE. The effect of the remained risk factors was thoroughly evaluated, and finally, a model integrating age, Na^+^, SOFA, heart rate, and body temperature was established. The nomogram was plotted to predict the probability of the incidence of SAE (Figure 3). The predictive capacity of the nomogram model was demonstrated through ROC analysis in both the training set and the validation set. The AUCs were 80.2% in the training set and 80.9% in the validation set (Figures 4A, B).

### 3.5. Validation of the prediction nomogram

**Figure 5:**
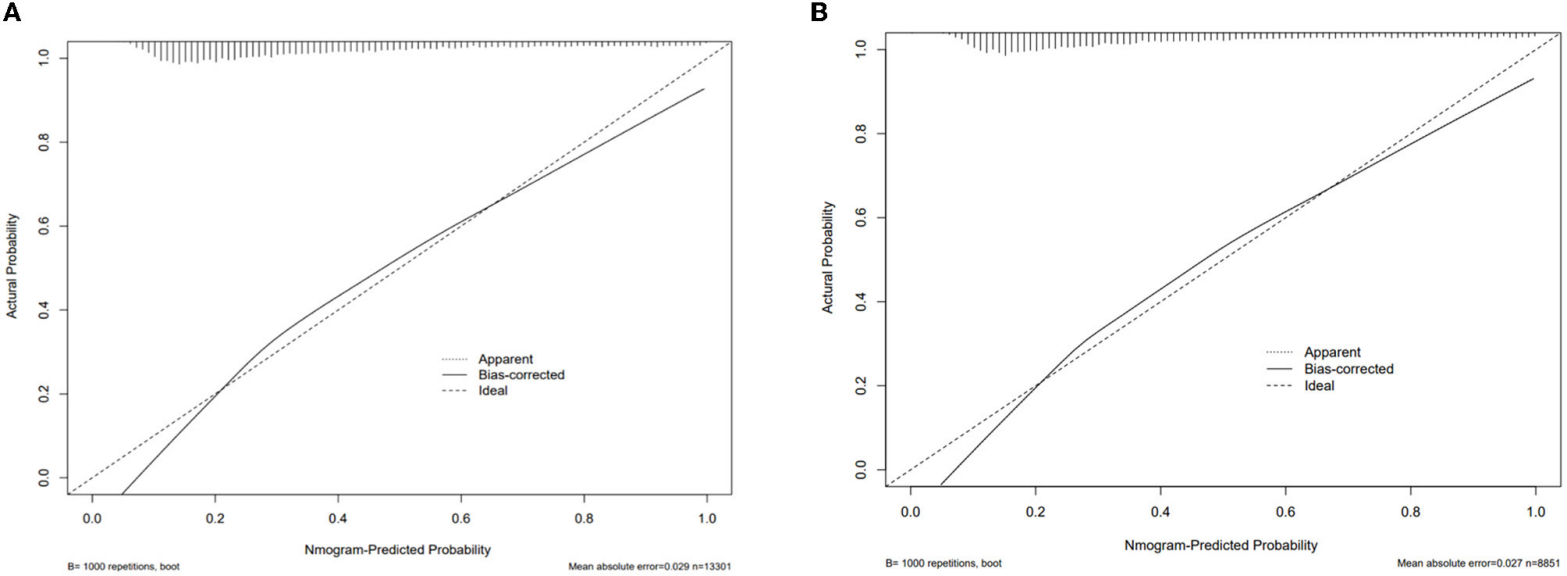
Calibration curve analysis of the nomogram. **(A)** in training set, **(B)** in validation set.

A calibration curve analysis was used to demonstrate the clinical accuracy of the prediction nomogram. Calibration curves were plotted in both the training and validation sets, and the bias-corrected line was formed using a bootstrap method. In both sets, the bias-corrected curve and apparent curve slightly deviated from the reference line, but a good consistency between observation and ideal reference was still observed (Figures 5A, B). A decision curve analysis (DCA) was performed to demonstrate the net benefit achieved through the clinical use of the nomogram. The results showed the nomogram gained clinical net benefit both in the training set and validation set (Supplementary material 2A, B).

### 3.6. Patients with higher SOFA or more advanced age had a higher incidence of SAE

**Figure 6:**
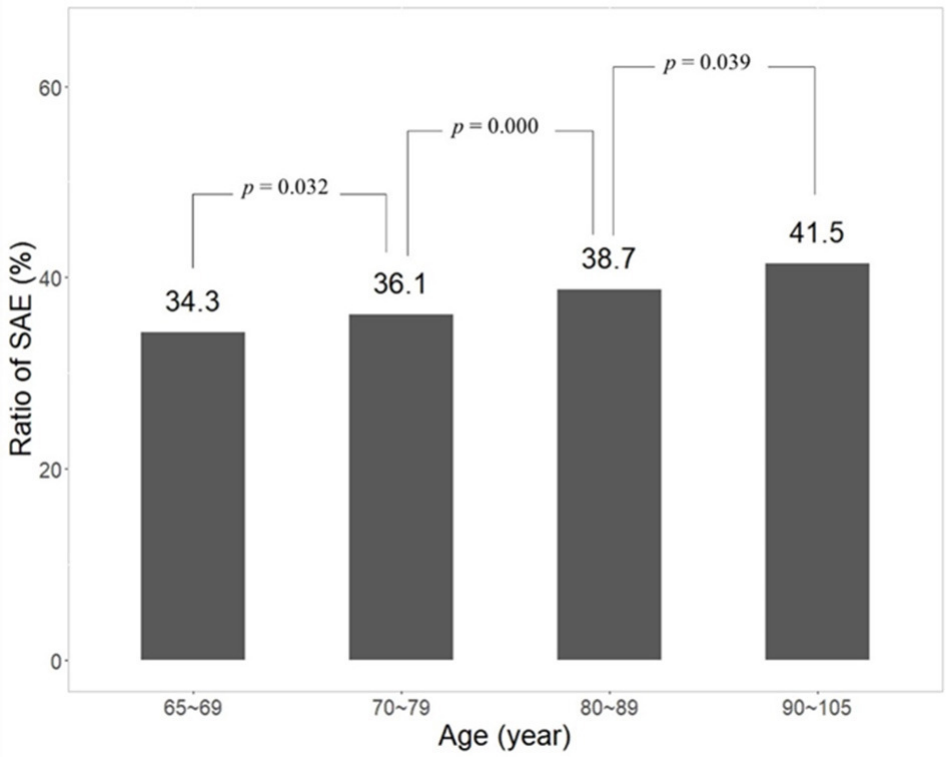
Significant increase of SAE incident related to greater age.

**Figure 7:**
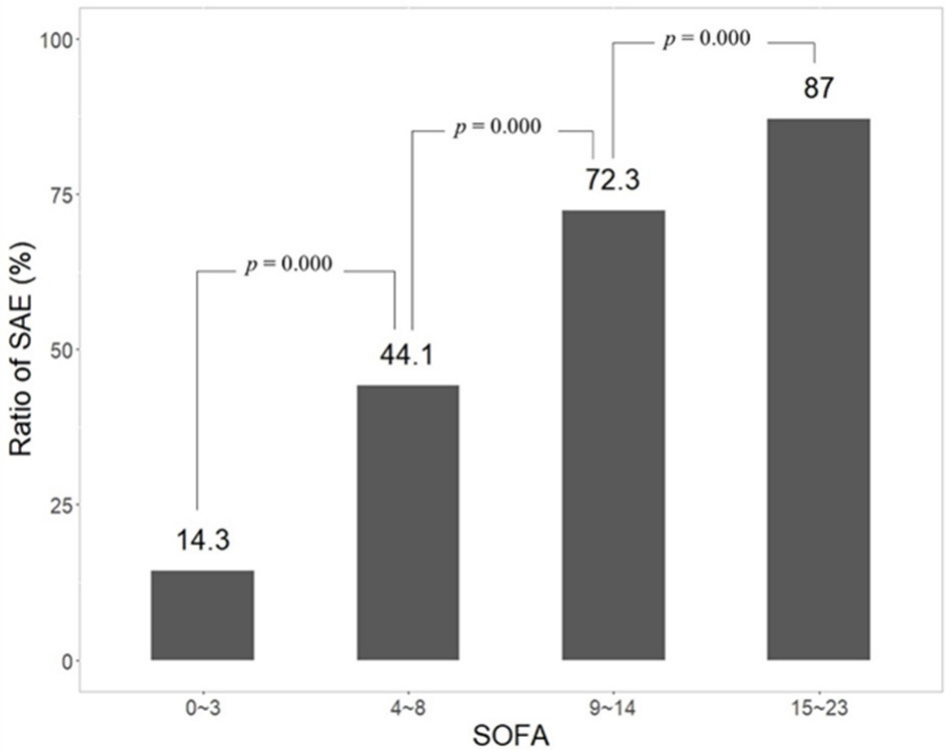
Significant increase of SAE incidence related to higher SOFA.

To demonstrate the influence of age and SOFA on the incidence of SAE, patients were divided into different groups according to age or SOFA. Data showed that the incidence of SAE increased statistically as the age of patients became greater (Figure 6, Supplementary material 3) (*p* = 0.032 between 65~69 and 70~79; *p* = 0.000 between 70~79 and 80~89; *p* = 0.039 between 80~89 and 90~105). In the other results, patients were divided into four groups in terms of SOFA (0~3, 4~8, 9~14, and 15~23). The incidence of SAE increased steadily from 14.3, 44.1, and 72.3 to 87.0%. There was a significant difference between groups of SOFA (*p* < 0.001) (Figure 7, Supplementary material 4).

## 4. Discussion

Currently, there is a shortage of data concerning SAE in elderly patients. The present study provides data about the morbidity of SAE and demonstrates that SAE is an independent risk factor of in-hospital mortality in elderly patients in the ICU. Furthermore, this study identifies relevant risk factors of SAE and develops a prediction nomogram as a useful tool for the clinical management of SAE in elderly patients.

Sepsis is still a crucial cause of death in critical illnesses. More than 19 million patients with sepsis were diagnosed and approximately 5.3 million patients died of it globally every year (15). It is important to note that cognitive impairment is a common clinical syndrome in patients with sepsis, especially in the elderly population. SAE is a neurological dysfunction of the central nervous system induced by sepsis (16). Previous studies reported that 30–70% of patients with sepsis developed SAE (17). A study enrolled 821 patients with respiratory failure or shock in the medical or surgical intensive care unit (ICU). The median of SOFA was 9 (7, 12) (expressed as median and quartile). A total of 74% of patients developed delirium, which indicated that patients in the ICU were at high risk of cognitive impairment (18). The other study enrolled 175 patients with sepsis, of which 74 patients (42.3%) were diagnosed with SAE. The average age was approximately 56.2 ± 15 years in this study (19). In the present study, as shown in Table 1, there were 8,290 patients with SAE and the incidence of SAE in patients with sepsis was 77.5%. This result (77.5%) was much higher than the data reported previously concerning the less elderly population, which indicated that elderly patients were more vulnerable to developing SAE.

SAE is a reflection of critical illness in the central nervous system. It is the neurological dysfunction induced by the combination of extracranial infection. Different peripheric cytokines, immune cells, or even bacteria infiltrate the cerebrum and take part in the inflammation injury process of the brain. Inflammatory and immunologic injuries play important roles in it. Astrocytes and microglial cells in the brain are activated and also contribute to the development of SAE (7, 20). The decrease in consciousness level is the most common result of SAE. GCS evaluation is reported to be a validated pragmatic way to assess neurological injury in the brain. This was why the definition of SAE in the present study was based on GCS evaluation at ICU admission (21). Delirium is also an important clinical manifestation of SAE and is taken as the other factor in SAE's definition. Therefore, the definition of SAE in the present study was based on GCS and delirium, which was also reported in previous studies (10, 14). SAE is reported to be associated with increased mortality, prolonged length of ICU stay, and hospital stay. These were compared with the results in the present study. The mortality in patients with SAE was much higher than that in patients without SAE (18.8 vs. 8.9%, *p* < 0.001). Patients with SAE had a longer length of ICU stay (4.94 ± 5.50 vs. 3.30 ± 4.30 days, *p* < 0.001) and hospital stay (12.05 ± 12.37 vs. 9.06 ± 8.97 days, *p* = 0.000). A previous study [16] reported the 28-day mortality in SAE and non-SAE groups (45.95 vs. 17.82%, *p* < 0.01). The average of SOFA was approximately 9.1 ± 4.8 in this study. However, in our study, the average of SOFA was 5.04 ± 3.35. It may be the reason why the mortality in our study is lower. The other study based on MIMIC-IV reported 17.62% of patients with SAE died in 30 days which was comparable with our results (22).

SOFA is a classical tool in the evaluation of the severity of illnesses and has a good capability in the prediction of prognosis (23). SOFA was proven to be positively related to the occurrence of delirium (24, 25). In the present study, SOFA was associated with the occurrence of SAE significantly (*p* < 0.001) and showed a strong influence in the nomogram model. The incidence of SAE rose steadily as SOFA increased (*p* < 0.001) (Figure 7). Age is also an independent risk factor of SAE. In elderly patients in ICU, delirium was reported to be more prevalent (26, 27). Our data showed that the incidence of SAE increased statistically from 34.3 to 41.5% as the age of patients rose from 65~69 to 90~69 years (*p* < 0.05). Hypernatremia is a common metabolic disturbance. A previous study showed hypernatremia was associated with SAE significantly (hypernatremia >145 mmol/l, OR = 2.30, 95% CI: 1.48–3.57 by logistic regression analysis) [10]. In the present study, hypernatremia was related to SAE significantly which was proved through logistic regression analysis (OR = 1.028, 95%CI: 0.021–0.034). So, Na^+^ was adopted in the nomogram model. Platelet was not included in the nomogram because there was little clinical correlation between it and delirium. Tachycardia and high body temperature were also reported to be associated with delirium and impairment of cognition. A multi-centric benchmark study from Lebanon reported the prevalence and incidence of delirium in elderly patients (over 65 years). Tachycardia is a common clinical manifestation induced by fever, hypoxia, and shock. According to this consideration, many patients with delirium were accompanied by tachycardia. Tachycardia is related to cognitive impairment indirectly. Fever usually indicates systemic infection and is a criterion of sepsis. Fever itself could disturb the metabolism of the brain and results in neurological disorder (28). Heart rate and body temperature were integrated into the nomogram and affected the prediction of the nomogram significantly in the present study. Tachycardia and fever usually indicate a severe condition of illnesses, which are associated with a higher risk of SAE.

All the laboratory tests adopted in the nomogram were taken from the elderly patients at admission to the ICU, as well as the vital signs and SOFA score. Clinicians need to collect the data in the first 24 h after ICU admission to carry on the calculation of the nomogram. This is time-consuming for busy clinical work. Next, the nomogram will be integrated into the hospital information system. The risk of SAE occurrence could be calculated and shown automatically.

The present study has several limitations. First, data were extracted from MIMIC-IV, which made this study a retrospective cohort study from a single center. Therefore, bias could not be avoided. In the real world, it is unavoidable to have occurrence bias in the epidemic investigation of some particular disease in a selected population. The setting is based on the elderly population in ICU. However, there was no intentional intervention in the enrollment, and a total of 22,361 patients were enrolled. The large sample size helped to decrease the occurrence bias to an acceptable level. The other limit is that the definition of SAE was based on delirium and GCS scores. This definition is not as strict as the diagnosis of SAE based on particular clinical evaluation or physico-chemical inspection. All these may reduce the generalizability of the results. A prospective, multiple-center, randomized control trial based on specialized examination is needed in future.

## 5. Conclusion

Sepsis-associated encephalopathy is a high-incidence complication of sepsis in elderly patients in the ICU. SAE is an independent risk of in-hospital mortality. The nomogram integrating several key clinical symptoms and scores is efficient in predicting SAE, which helps in optimizing the management of SAE for elderly patients in the ICU.

## Data availability statement

The original contributions presented in the study are included in the article/Supplementary material, further inquiries can be directed to the corresponding author.

## Ethics statement

This study has been approved by the Ethics Committee of the Chinese PLA General Hospital (number: S2017–054-01).

## Author contributions

QZ and JX took part in the study design, performed statistical analyses, and drafted the manuscript. XL had access permission to MIMIC-IV and extracted data for the present study. HL conceived the study, participated in the design, and revised the manuscript critically for important intellectual content. All authors read and approved the final manuscript.

## References

[1] Ely EW Shintani A Truman B Speroff T Gordon SM Harrell FE. Delirium as a predictor of mortality in mechanically ventilated patients in the intensive care unit. JAMA. (2004) 291:1753–62. 10.1001/jama.291.14.1753 15082703

[2] Polito A Eischwald F Maho AL Polito A Azabou E Annane D. Pattern of brain injury in the acute setting of human septic shock. Crit Care Lond Engl. (2013) 17:R204. 10.1186/cc12899 24047502 PMC4057119

[3] Azabou E Magalhaes E Braconnier A Yahiaoui L Moneger G Heming N. Early standard electroencephalogram abnormalities predict mortality in septic intensive care unit patients. PLoS ONE. (2015) 10:e0139969. 10.1371/journal.pone.0139969 26447697 PMC4598037

[4] Gilmore EJ Gaspard N Choi HA Cohen E Burkart KM Chong DH. Acute brain failure in severe sepsis: a prospective study in the medical intensive care unit utilizing continuous EEG monitoring. Intensive Care Med. (2015) 41:686–94. 10.1007/s00134-015-3709-1 25763756

[5] Hatch R Young D Barber V Griffiths J Harrison DA Watkinson P. Anxiety, depression and post traumatic stress disorder after critical illness: a UK-wide prospective cohort study. Crit Care Lond Engl. (2018) 22:310. 10.1186/s13054-018-2223-6 30466485 PMC6251214

[6] Righy C Rosa RG da Silva RT Kochhann R Migliavaca CB Robinson CC. Prevalence of post-traumatic stress disorder symptoms in adult critical care survivors: a systematic review and meta-analysis. Crit Care Lond Engl. (2019) 23:213. 10.1186/s13054-019-2489-3 31186070 PMC6560853

[7] Mazeraud A Righy C Bouchereau E Benghanem S Bozza FA Sharshar T. Septic-associated encephalopathy: a comprehensive review. Neurotherapeutics. (2020) 17:392–403. 10.1007/s13311-020-00862-1 32378026 PMC7283452

[8] Wu WT Li YJ Feng AZ Li L Huang T Xu AD. Data mining in clinical big data: the frequently used databases, steps, and methodological models. Mil Med Res. (2021) 8:44. 10.1186/s40779-021-00338-z 34380547 PMC8356424

[9] Yang Y Liang S Geng J Wang Q Wang P Cao Y. Development of a nomogram to predict 30-day mortality of patients with sepsis-associated encephalopathy: a retrospective cohort study. J Intensive Care. (2020) 8:45. 10.1186/s40560-020-00459-y 32637121 PMC7331133

[10] Yang J Li Y Liu Q Li L Feng A Wang T. Brief introduction of medical database and data mining technology in big data era. J Evid Based Med. (2020) 13:57–69. 10.1111/jebm.12373 32086994 PMC7065247

[11] Cavaillon JM Chrétien F. (2019). From septicemia to sepsis 3.0-from Ignaz Semmelweis to Louis Pasteur. Genes Immun. 20:371–82. 10.1038/s41435-019-0063-2 30903106

[12] Ely EW Margolin R Francis J May L Truman B Dittus R. Evaluation of delirium in critically ill patients: validation of the confusion assessment method for the intensive care unit (CAM-ICU). Crit Care Med. (2001) 29:1370–9. 10.1097/00003246-200107000-00012 11445689

[13] Sonneville R de Montmollin E Poujade J Garrouste-Orgeas M Souweine B Darmon M. Potentially modifiable factors contributing to sepsis-associated encephalopathy. Intensive Care Med. (2017) 43:1075–84. 10.1007/s00134-017-4807-z 28466149

[14] Chen J Shi X Diao M Jin G Zhu Y Hu W. A retrospective study of sepsis-associated encephalopathy: epidemiology, clinical features, and adverse outcomes. BMC Emerg. Med. (2020) 20:77. 10.1186/s12873-020-00374-3 33023479 PMC7539509

[15] Rudd KE Johnson SC Agesa KM Shackelford KA Tsoi D Kievlan DR. Global, regional, and national sepsis incidence and mortality, 1990–2017: analysis for the global burden of disease study. Lancet. (2020) 395:200–11. 10.1016/S0140-6736(19)32989-7 31954465 PMC6970225

[16] Mazeraud A Bozza FA Sharshar T. Sepsis-associated encephalopathy is septic. Am J Respir Crit Care Med. (2018) 197:698–9. 10.1164/rccm.201712-2593ED 29360405

[17] Tauber SC Djukic M Gossner J Eiffert H Brück W Nau R. Sepsis-associated encephalopathy and septic encephalitis: an update. Expert Rev Anti Infect Ther. (2021) 19:215–31. 10.1080/14787210.2020.1812384 32808580

[18] Pandharipande PP Girard TD Jackson JC Morandi A Thompson JL Pun BT. Long-term cognitive impairment after critical illness. N Engl J Med. (2014) 369:1306–16. 10.1056/NEJMoa1301372 24088092 PMC3922401

[19] Feng Q Ai YH Gong H Wu L Ai ML Deng SY. Characterization of sepsis and sepsis-associated encephalopathy. J Intensive Care Med. (2019) 34:938–45. 10.1177/0885066617719750 28718340

[20] Ren C Yao RQ Zhang H Feng YW Yao YM. Sepsis-associated encephalopathy: a vicious cycle of immunosuppression. J Neuroinflammation. (2020) 17:14. 10.1186/s12974-020-1701-3 31924221 PMC6953314

[21] Sharshar T Citerio G Andrews PJ Chieregato A Latronico N Menon DK. Neurological examination of critically ill patients: a pragmatic approach. Report of an ESICM expert panel. Intensive Care Med. (2014) 40:484–95. 10.1007/s00134-014-3214-y 24522878

[22] Peng L Peng C Yang F Wang J Zuo W Cheng C. Machine learning approach for the prediction of 30-day mortality in patients with sepsis-associated encephalopathy. BMC Med Res Methodol. (2022) 22:183. 10.1186/s12874-022-01664-z 35787248 PMC9252033

[23] Koch C Edinger F Fischer T Brenck F Hecker A Katzer C. Comparison of qSOFA score, SOFA score, and SIRS criteria for the prediction of infection and mortality among surgical intermediate and intensive care patients. World J Emerg Surg. (2020) 15:63. 10.1186/s13017-020-00343-y 33239088 PMC7687806

[24] Rahimi-Bashar F Abolhasani G Manouchehrian N Jiryaee N Vahedian-Azimi A Sahebkar A. Incidence and risk factors of delirium in the intensive care unit: a prospective cohort. Biomed Res Int. (2021) 2021:6219678. 10.1155/2021/6219678 33506019 PMC7810554

[25] Liu H Zhao Q Liu X Hu X Wang L Zhou F. Incidence and interaction factors of delirium as an independent risk of mortality in elderly patients in the intensive units: a retrospective analysis from MIMIC-IV database. Aging Clin Exp Res. (2022) 34:2865–72. 10.1007/s40520-022-02215-8 36057682

[26] Bugiani O. Why is delirium more frequent in the elderly? Neurol Sci. (2021) 42:3491–503. 10.1007/s10072-021-05339-3 34031797 PMC8143064

[27] Kirfel A Menzenbach J Guttenthaler V Feggeler J Mayr A Coburn M. Postoperative delirium after cardiac surgery of elderly patients as an independent risk factor for prolonged length of stay in intensive care unit and in hospital. Aging Clin Exp Res. (2021) 33:3047–56. 10.1007/s40520-021-01842-x 33813686 PMC8595147

[28] Sun G Qian S Jiang Q Liu K Li B Li M. Hyperthermia-induced disruption of functional connectivity in the human brain network. PLoS ONE. (2013) 8:e61157. 10.1371/journal.pone.0061157 23593416 PMC3620175

