# Supplementary material for: The nomogram to predict the occurrence of sepsis-associated encephalopathy in elderly patients in the intensive care units: A retrospective cohort study
Source: Front Neurol. 2023 Feb 2;14:1084868. doi: 10.3389/fneur.2023.1084868 (PMC9932587; doi:10.3389/fneur.2023.1084868)
Supplement: Supplementary file 1 [file Table_1.docx]

Supplementary file 1. baseline characteristics and outcome data between training set and validation set

| characteristics | all | Training set (60%) | Validation set (40%) | *p* value |
| --- | --- | --- | --- | --- |
| number | 22361 | 13416 | 8945 |  |
| Age (year) | 77.25±8.05 | 77.23±8.01 | 77.28±8.12 | 0.650 |
| Female/male | 10341/12020 | 6209/7207 | 4132/4813 | 0.516 |
| Hemoglobin (g/dL) | 10.05±2.11 | 10.03±2.11 | 10.08±2.12 | 0.083 |
| WBC (10^9^/L) | 14.28±11.83 | 14.18±6.83 | 14.23±6.64 | 0.893 |
| Platelet (10^9^/L) | 186.92±94.91 | 186.58±94.77 | 187.42±95.66 | 0.523 |
| Creatinine (mg/mL) | 1.49±1.35 | 1.49±1.35 | 1.49±1.36 | 0.967 |
| Glucose (mg/dL) | 165.10±91.30 | 164.50±89.03 | 166.00±95.64 | 0.231 |
| Na^+^ (mmol/L) | 139.81±4.95 | 139.84±4.94 | 139.77±4.99 | 0.260 |
| PaO_2_ (mmHg) | 115.13±50.74 | 115.10±68.83 | 115.17±70.18 | 0.567 |
| Heart rate (beat/minute) | 82.40±14.93 | 82.36±14.97 | 82.44±14.87 | 0.693 |
| Mean artery pressure (mmHg) | 76.89±10.20 | 76.85±10.17 | 76.96±10.24 | 0.420 |
| Respiratory rate (time/minute) | 19.17±3.57 | 19.17±3.56 | 19.17±3.58 | 0.924 |
| Body tempreture (℃) | 36.77±0.48 | 36.78±0.48 | 36.77±0.49 | 0.297 |
| SOFA | 5.04±3.35 | 4.26±3.07 | 5.04±3.37 | 0.960 |
| Sepsis (count/percent) | 10694/47.8% | 6386/47.6% | 4308/48.2% | 0.418 |
| Delirium (number/percent) | 5453/24.4% | 3262/24.3% | 2191/24.5% | 0.771 |
| shock (count/percent) | 4370/19.5% | 2594/19.3% | 1776/19.9% | 0.346 |
| SAE (count/percent) | 8290/37.1% | 4950/36.9% | 3340/37.3% |  |
| Outcome data |  |  |  |  |
| ICU days (day) | 3.85±4.79 | 3.92±5.03 | 3.84±4.91 | 0.079 |
| Hospital days (day) | 10.06±10.32 | 10.02±10.37 | 9.98±10.45 | 0.098 |
| In-hospital mortality(count/percent) | 2809/12.7% | 1687/12.6% | 1122/12.5% | 0.992 |

* *p* <0.05, ***p* <0.01. The values were shown as mean ± SD.
