# Supplementary material for: The nomogram to predict the occurrence of sepsis-associated encephalopathy in elderly patients in the intensive care units: A retrospective cohort study
Source: Front Neurol. 2023 Feb 2;14:1084868. doi: 10.3389/fneur.2023.1084868 (PMC9932587; doi:10.3389/fneur.2023.1084868)
Supplement: Supplementary file 2 [file Table_2.DOCX]

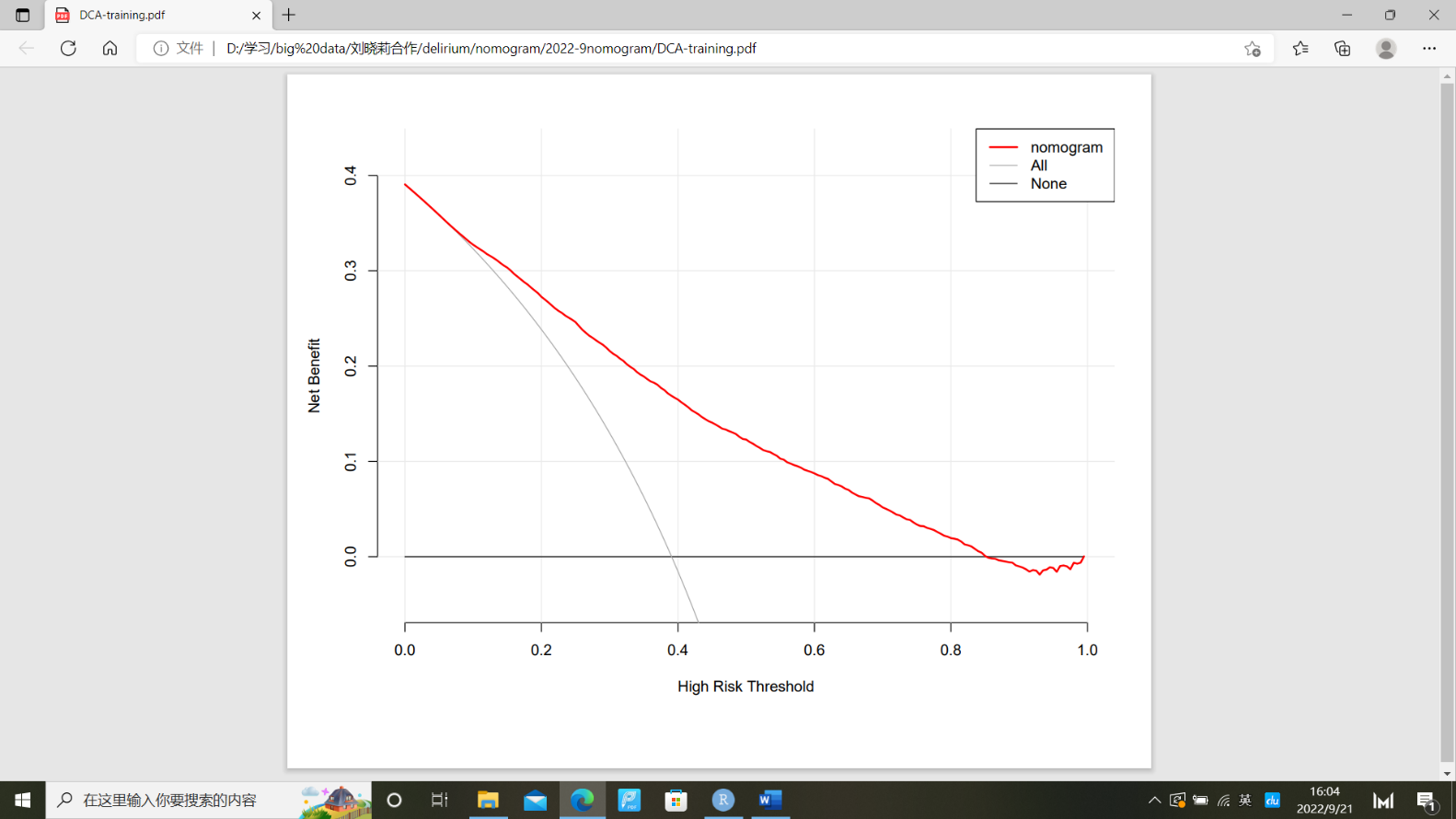


2 A. In training set


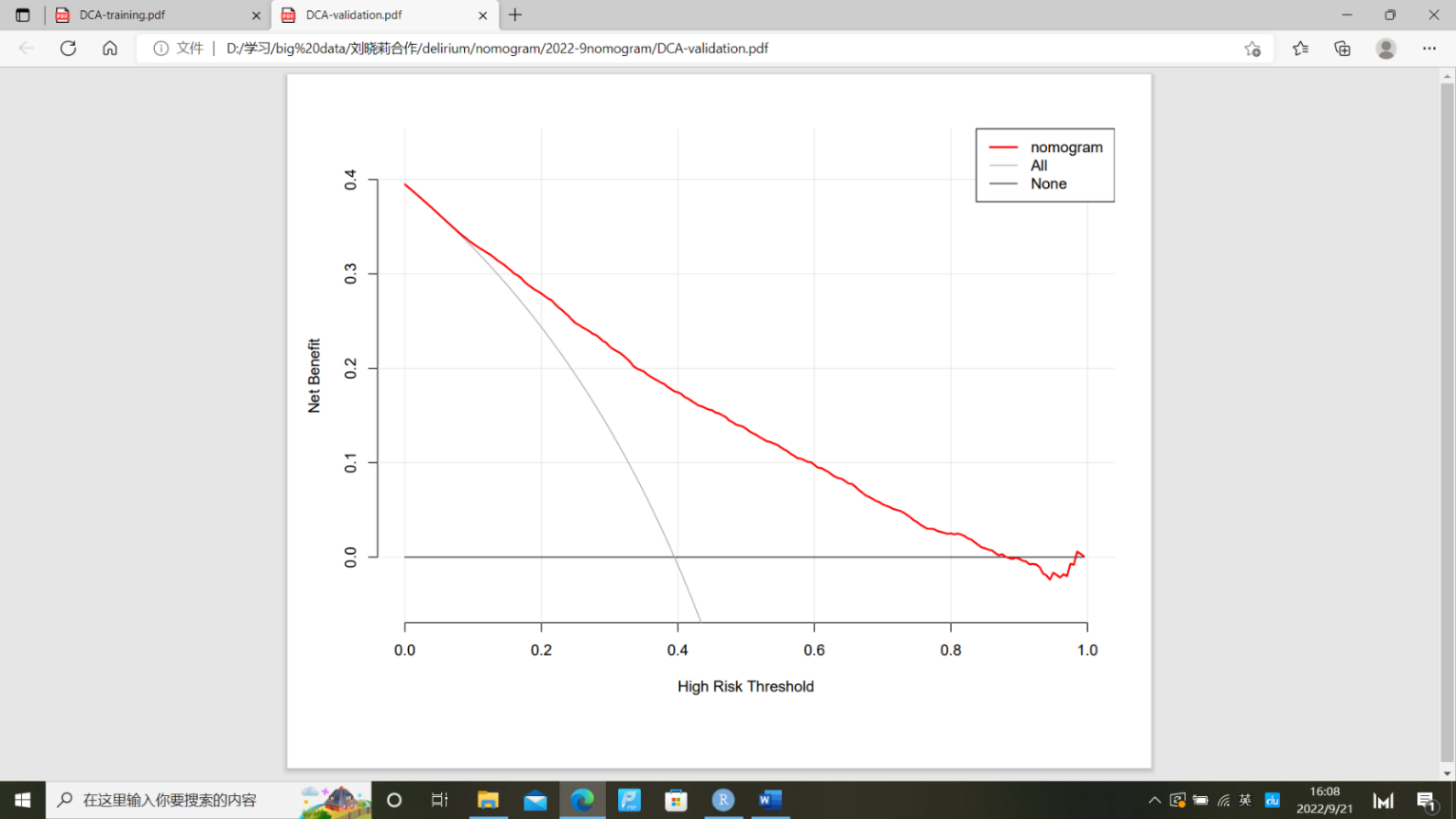


2B. In validation set

Supplementary file 2. Decision curve analysis of the nomogram 2A, in training set; 2B, in validation set
