# Supplementary material for: The nomogram to predict the occurrence of sepsis-associated encephalopathy in elderly patients in the intensive care units: A retrospective cohort study
Source: Front Neurol. 2023 Feb 2;14:1084868. doi: 10.3389/fneur.2023.1084868 (PMC9932587; doi:10.3389/fneur.2023.1084868)
Supplement: Supplementary file 3 [file Table_3.DOCX]

| groups | Age(year) | Patients | SAE | Non-SAE | Ratio of SAE | P value |
| --- | --- | --- | --- | --- | --- | --- |
| 1 | 65-69 | 4853 | 1664 | 3189 | 34.3% |  |
| 2 | 70-79 | 8754 | 3164 | 5590 | 36.1% | 0.032 (2 to 1) |
| 3 | 80-89 | 6648 | 2587 | 4061 | 38.7% | 0.000 (3 to 2) |
| 4 | 90-105 | 2106 | 875 | 1231 | 41.5% | 0.039 (4 to 3) |
| summary | 65-105 | 22361 | 8290 | 14071 | 37.1% |  |

Supplementary file 3. significant increase of SAE incidence related to greater age
