# Supplementary material for: The nomogram to predict the occurrence of sepsis-associated encephalopathy in elderly patients in the intensive care units: A retrospective cohort study
Source: Front Neurol. 2023 Feb 2;14:1084868. doi: 10.3389/fneur.2023.1084868 (PMC9932587; doi:10.3389/fneur.2023.1084868)
Supplement: Supplementary file 4 [file Table_4.DOCX]

| groups | SOFA | Patients | SAE | Non-SAE | Ratio of SAE | P value |
| --- | --- | --- | --- | --- | --- | --- |
| 1 | 0-3 | 8549 | 1221 | 7328 | 14.3% |  |
| 2 | 4-8 | 10527 | 4645 | 5882 | 44.1% | 0.000 (2 to 1) |
| 3 | 9-14 | 2930 | 2115 | 815 | 72.3% | 0.000 (3 to 2) |
| 4 | 15-23 | 355 | 309 | 46 | 87.0% | 0.000 (4 to 3) |
| summary | 0-23 | 22361 | 8290 | 14071 | 37.1% |  |

Supplementary file 4. significant increase of SAE incidence related to higher SOFA
